# Organised Charity Abroad

**Published:** 1899-01-14

**Authors:** 


### ORGANISED CHARITY ABROAD.

### THE HOUSE OF THE BON SAUVEUR AT CAEN.—III.

It is very hard to get at the real principles upon which an institution is worked. Even with the rules and constitution before one, so much scope is left for feeling, sentiment, and sympathy. The French as a nation are fonder of community life than the English; a certain society air of conversational friendliness pervades the poorest of French institutions, and this is no doubt fostered to a large extent by the outdoor life it is possible to lead during so many out of the twelve months. The secret of the success of the Bon Sauveur seems to be in the clever application in detail of the psychological theory of the continuity and association of ideas. The patients are considered and treated as if they were sane—as far, of course, as is compatible with their safety—and the life inside the institution is the same as that of any other French town. Fits or paroxysms are regarded as unfortunate accidents, to be forgotten as soon as possible. Social intercourse is brought into prominence, and used as a safeguard against individual peculiarities. It is needless to say that all this is only rendered possible by the size of the gardens and grounds, and by the ample accommodation. In fairness to the Sisters, who devote their lives to this work, we must add that their unfailing cheerfulness, hopefulness, and "brotherly kindness" is beyond praise. They take a human personal interest in each of their patients, and a reciprocity of feeling is speedily established. There are, however, several points which the critical observer might wish to raise; as, for instance, whether the regulations as to entrance are really efficient—*e.g.*, whether it is possible to gain admittance, to get shut up in the Bon Sauveur on false pretences? In answer to this, it appears that in the case of French patients nothing of this kind can happen. The Government receives an account of the case of every individual, and uses its secret police in the most indirect way to confirm the certificate sent in by the patient's doctor. In the case of foreigners the patient has no such safeguard. Practically, however, in the case of English subjects the matter is brought under the immediate control of Her Majesty's Consul, who receives due intimation of all the details under which it is proposed to detain the individual, and who can and does interfere if he thinks he is called upon to do so. In spite of this, it would seem desirable to lay greater stress on the official declaration of the medical attendants. A certificate of insanity should be endorsed not only by the patient's personal adviser, but should be confirmed by other colleagues, and not by the doctor of the institution alone, as is at present the custom. So, too, in the event of a cure in the case of British subjects, the patient sends a written notification of his reasons for wishing to leave to the Consul, who visits him in person, and takes such steps as he may consider desirable. It is to be noted that specialists have taken the responsibility of detaining patients who have seemed to be perfectly sane to the ordinary observer. It will be seen that the responsibility falls greatly on the Consul, which is perhaps hardly fair. Of course, in the case of the majority of patients—those sent by the French Government—motives of economy are all on the side of dismissal. On an average 64 out of 166 cases are affected. Double registers are kept—one by the Préfet of the Department and one by the Bon Sauveur. These are revised monthly by the Préfet and by the Superior. Private patients are only allowed as there may be room for them, after the Government cases have been arranged for. George Brummel, the friend of King George IV., Bourrienne, the friend of Napoleon, and Destouches have been among the patients.

With regard to the deaf and dumb, statistics show that they are very equally distributed in France to the number of 12,000, the south having as many as the north. Many are scrofulous or lymphatic, and the affliction seems to be hereditary. It was found in the Department Calvados that six families produced fourteen deaf and dumb; in La Manche four families produced fourteen. Social position seems not to affect the question; privation or comfort do not touch the statistics. M. Jamet made these unfortunate people his special study, and much of his own personal attention went to the Department of the Bon Sauveur which is devoted to them.

In modern times the Spanish were the first to call attention to the need of special schools for deaf and dumb. A work on the subject by a Spanish monk was printed in 1606. M. Bonnet, tutor to the Connétable de Castille, who became deaf and dumb after his fourth year, published an "Arte para ensenar los Mudos Hablar" in 1620, which was acknowledged by the Academy of Madrid. The English Ambassador in Madrid studied this system, and books on the subject began to appear in England, 1655 and 1666. About 1670 four schoolmasters—Wally, Digby, Wallis, and Burnet—printed their methods. During the seventeenth century two Spanish monks actually undertook to teach the deaf and dumb. Conrad Amman, a physician born in Switzerland, who lived in Amsterdam, and died about 1800, wrote many works on the subject—"Surdus Loquens," "De Loqueta Surdorum et Mutorum," &c. MM. Pereires, Ernand, Heinrich Deschamps, and l'Abbé de l'Epée followed him. The German Franz von Helmont joined a band of Bohemians, roamed over Europe, and returned home to teach the deaf and dumb. While M. Vanin and the Abbé de l'Epée taught by the method of signs at Paris, the method of M. de l'Epée was established at Madrid, Zurich, Vienna, Amsterdam, and Rouen. A school was founded at Bordeaux, and another at Angers, Aurai Rhodez, Saint Etienne, Arras, London, Petersburg, and the United States.

To avoid needless technicalities, we may group the various methods in use in these schools under three headings: (1) Teacher to speak; (2) teacher to copy signs, dictation much used; (3) questions pupil, and obliges him to seek terms for an answer.

M. Jamet had a method of his own, founded on six fundamental principles: (1) Signs are not a language—they are merely the manual expression of words; (2) make the signs of words, and not of things; (3) signs must be simple; (4) signs must be invariable—only one sign for each word; (5) in however many senses a word may be used, it must have but one sign; (6) the preposition which enters into the composition of the various parts of a discourse must be indicated by signs. M. Jamet insisted strongly on the distinction between "language" and "langue"—"Les signs ne sont pas une langue." The sound of the voice which articulates words is not a language; it is only the pronunciation of the words of a language. In every "langue" we get (1) the written form, (2) the accepted sense, and (3) the sound. We can say, "La langue des gestes." Signs are "La pronunciation manuelle." Those signs which stand for words have, when used for the first time, to be taught and illustrated by pantomime.

The Bon Sauveur has remained almost unknown to the British medical world, but this summer it was visited by a member of the London County Council and by a committee of London doctors.

